# Metal-Free Graphitic Carbon Nitride Photocatalyst Goes Into Two-Dimensional Time

**DOI:** 10.3389/fchem.2018.00551

**Published:** 2018-12-10

**Authors:** Gang Zhao, Hongcen Yang, Mengqi Liu, Xijin Xu

**Affiliations:** Laboratory of Functional Micro-nano Materials and Devices, School of Physics and Technology, University of Jinan, Jinan, China

**Keywords:** two-dimensional g-C_3_N_4_, metal-free photocatalysts, atom doping, modification, heterojunction

## Abstract

Graphitic carbon nitride (g-C_3_N_4_) is always a research hotspot as a metal-free visible-light-responsive photocatalyst, in the field of solar energy conversion (hydrogen-production by water splitting). This critical review summarizes the recent progress in the design and syntheses of two-dimensional (2D) g-C_3_N_4_ and g-C_3_N_4_-based nanocomposites, covering (1) the modifications of organic carbon nitrogen precursors, such as by heat treatment, metal or metal-free atoms doping, and modifications with organic functional groups, (2) the influencing factors for the formation of 2D g-C_3_N_4_ process, including the calcination temperature and protective atmosphere, etc. (3) newly 2D g-C_3_N_4_ nanosheets prepared from pristine raw materials and bulk g-C_3_N_4_, and the combination of 2D g-C_3_N_4_ with other 2D semiconductors or metal atoms as a cocatalyst, and (4) the structures and characteristics of each type of 2D g-C_3_N_4_ systems, together with their optical absorption band structures and interfacial charge transfers. In addition, the first-principles density functional theory (DFT) calculation of the g-C_3_N_4_ system has been summarized, and this review provides an insightful outlook on the development of 2D g-C_3_N_4_ photocatalysts. The comprehensive review is concluded with a summary and future perspective. Moreover, some exciting viewpoints on the challenges, and future directions of 2D g-C_3_N_4_ photocatalysts are discussed and highlighted in this review. This review can open a new research avenue for the preparation of 2D g-C_3_N_4_ photocatalysts with good performances.

## Introduction

The energy crisis has become a growing concern as society continues to develop, which further necessitates the development of sustainable energy sources to supersede traditional fossil fuels (Chang et al., 2017; He et al., 2017a,b, 2018; Wang et al., 2018; Zhang G. G. et al., 2018). The hydrogen produced by the photocatalytic water splitting reaction under sunlight, resulting in solar-to-chemical energy conversion, has been deemed to play a key role in resolving the solar-to-chemical energy conversion (Zhong et al., 2016; Zhang G. G. et al., 2018; Zhang S. W. et al., 2018). As a half reaction of the hydrogen production via water splitting, the reaction progress is the decrease of protons/water to hydrogen (Bard and Fox, 1995; Zou et al., 2001). Although the produced hydrogen process refers to the simple reactants, demanding only two electrons to generate a hydrogen molecule, the reaction kinetics is slow due to the large energy barriers in the multiple reaction steps (Tu et al., 2013; Wondraczek et al., 2015; Zhang N. et al., 2015; Zhang et al., 2017a). At the same time, the reduction of water to hydrogen requires many photo-induced holes with oxidant properties (Zhang et al., 2014). Therefore, photocatalysts are indispensable for these reactions, which can generate photo-induced electrons and holes under sunlight. So far, the most effective photocatalysts are still metal-based materials (Ma F. K. et al., 2016; Ma Z. et al., 2016; Ai et al., 2018). However, the high cost and heavy-metal-toxicity of these photocatalysts limit their usage.

In recent decades, abundant non-metal photocatalysts, mainly based on earth-abundant non-metals elements (P, S, N, and C), have been explored. Among them is graphitic carbon nitride (g-C_3_N_4_), which has recently been widely used in the field of photocatalytic water splitting, as a metal-free and environmentally friendly photocatalytic material, (Wang et al., 2012, 2014; Low et al., 2014; Dong and Cheng, 2015), of which the bulk and granulated g-C_3_N_4_ are the most widely used electrocatalysts for hydrogen production. This seriously inhibits the efficiency of photocatalytic water splitting (Zhang G. G. et al., 2016). Moreover, the application of g-C_3_N_4_ is restricted in the reaction because of its frequent photo-corrosion under sunlight. Therefore, continuous efforts have been made to develop more stable and efficient g-C_3_N_4_-based heterogeneous photocatalysts in recent years (Wang et al., 2011; Shi et al., 2015; Li G. et al., 2016; Yang et al., 2016). Additionally, as a new type of two-dimensional (2D) material, 2D g-C_3_N_4_ has been utilized as a photocatalyst in solar-driven water splitting. The progress in this research field is discussed in this review.

This critical review summarizes the recent progress made in the formation of 2D g-C_3_N_4_ (g-C_3_N_4_-based nanocomposites) for hydrogen production, and further elucidates the modifications of functional groups, the influencing factors of the formation process, new methods, heterojunction nanostructures, and so on. In addition, the DFT calculations for the g-C_3_N_4_ systems are also summarized to provide an insightful outlook. Finally, this review is concluded with a summary and future perspective.

## Modifications of Carbon Nitride

As a fascinating material, 2D g-C_3_N_4_ has attracted worldwide attention (Ma et al., 2014; Liang et al., 2015), and promises access to a wide field of applications compared with other photocatalytic materials, due to its outstanding features, such as its non-metal and non-toxicity (Liu G. et al., 2015; Zhang G. G. et al., 2015; Zhang M. et al., 2016; Zhang et al., 2017b). Furthermore, g-C_3_N_4_ is a wide-band gap indirect semiconductor (Schwinghammer et al., 2013) with an appealing electronic structure. This allows its direct use as a heterogeneous photocatalyst. However, the photocatalytic effect of pure g-C_3_N_4_ is inferior to those of metal semiconductor photocatalysts. Therefore, some modifications, such as metal-free or metal atom doping, are necessary to improve the photocatalytic effects of g-C_3_N_4_.

**Figure 1:**
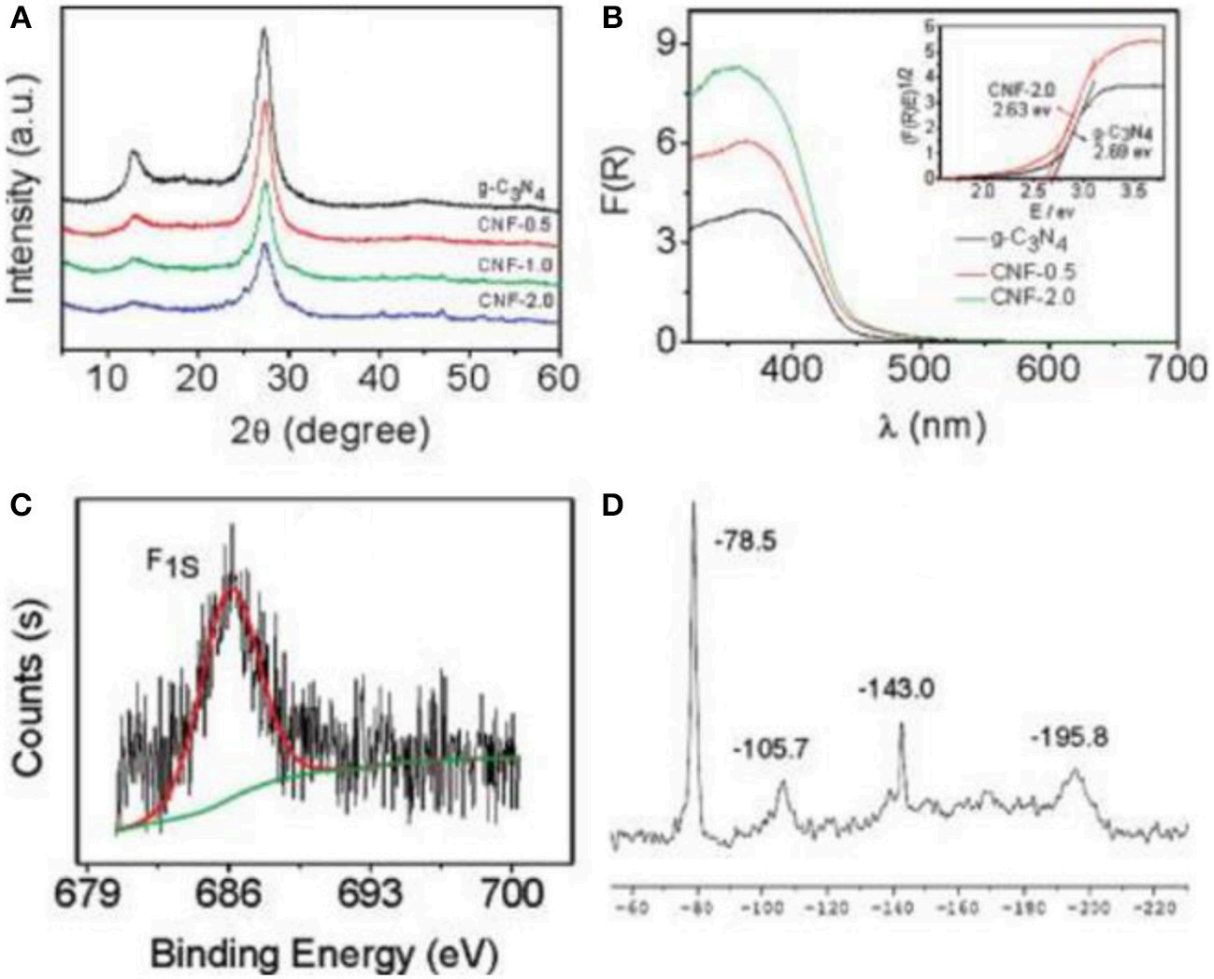
**(A)** XRD patterns of samples. **(B)** UV-Vis spectra of g-C_3_N_4_ and CNF-x (inset shows optical band gaps(E)_g_ of g-C_3_N_4_ and sample-2.0). **(C)** XPS spectrum of sample-2.0. **(D)** Solid-state MAS-NMR spectrum of sample-2.0 (Wang et al., 2010). Copyright 2010, American Chemical Society.

**Figure 2:**
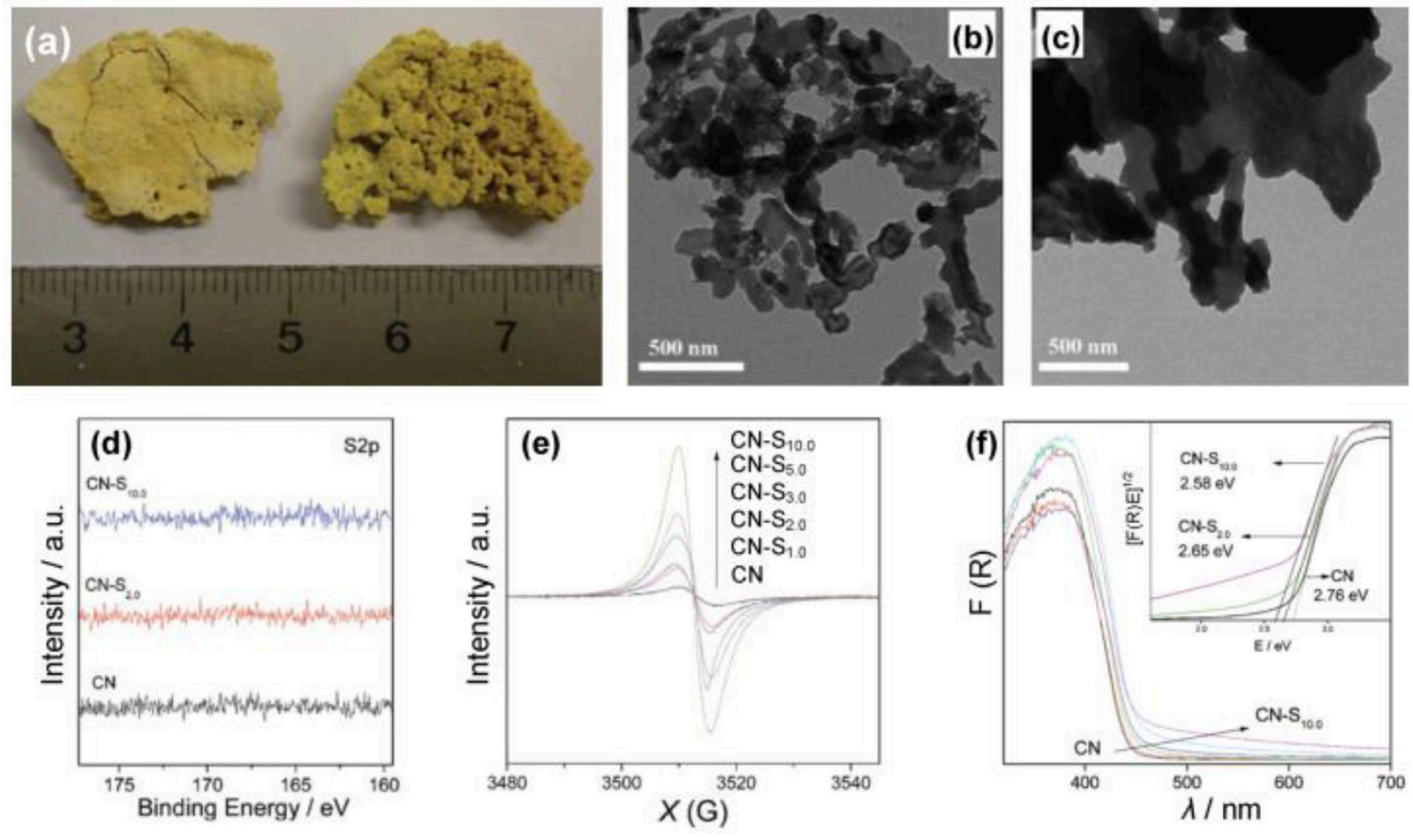
**(a)** Image of the g-C_3_N_4_ and CN-S_2.0_. **(b)** TEM images of CN-S_2.0_ and **(c)** unmodified g-C_3_N_4_. **(d)** High resolution XPS spectra of samples. **(e)** EPR spectra of samples in the dark. **(F)** UV–Vis spectra of samples (Zhang J. et al., 2012). Copyright 2012, American Chemical Society.

For the doping of g-C_3_N_4_ with metal-free atoms, halogen elements are very important and effective (Groenewolt and Antonietti, 2005; Chang et al., 2015; Han et al., 2016; Ye et al., 2016; Ma et al., 2017), The ionic radii of the incorporated guests of halogen elements decrease in the order F<Cl<Br (Chong et al., 2013). Generally, by using a heating treatment (dicyandiamide) in eutectic melting salt, such as LiY and KY (Y = F, Cl or Br), bulk g-C_3_N_4_ can chemically and physically be exfoliated into thin layers (Li^+^, K^+^ or X^−^) (Bojdys et al., 2013; Ma et al., 2017). As early as 2010, Wang et al. reported the synthesis of a fluorinated polymeric carbon nitride, which was employed as a heterogeneous catalyst for hydrogen generation from water. In addition, it was also used for the oxygenation of benzene into phenol under visible light (Figure 1, Wang et al., 2010). Other metal-free atoms are also used to dope g-C_3_N_4_, such as O, C, N, P, S, and B (Ran et al., 2015; Feng et al., 2016; Lu et al., 2017; Zhu et al., 2017). Zhang et al. used S8 (elemental sulfur) and melamine as the raw materials to obtain g-C_3_N_4_-S_x_, where x refers to the quality of S8 (Zhang J. et al., 2012), in which the absorption edges of CN-S_x_ samples became marginally red-shifted with adding S8 contents (Figure 2), thus decreasing the corresponding band gaps (E_g_) from 2.76 to 2.58 eV (Zhang J. et al., 2012).

**Figure 3:**
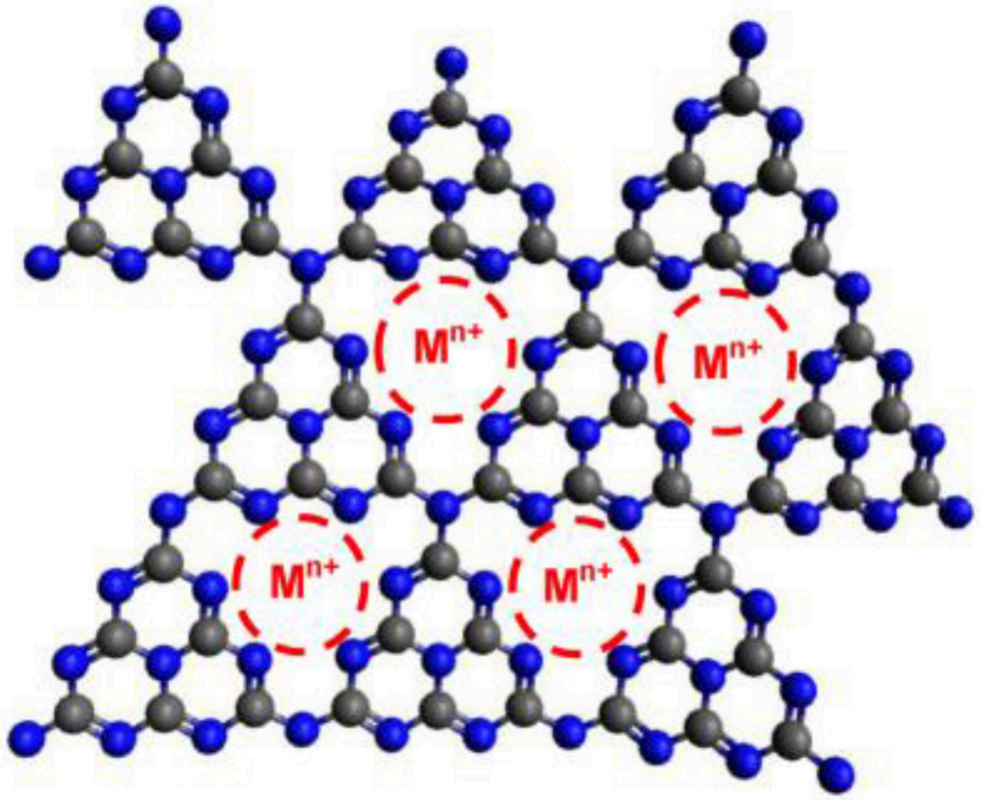
Schematic structure of g-C_3_N_4_ framework with obtaining metal ion (Ong et al., 2016). Copyright 2016, American Chemical Society.

The doping of g-C_3_N_4_ with metal atoms (Fe^3+^, Co^2+^, Ni^2+^, Cu^2+^, Zn^2+^, K^+^, Na^+^, and Li^+^) has also been widely used to enhance the catalytic properties of g-C_3_N_4_ (Figure 3, Pan et al., 2011; Yue et al., 2011; Ding et al., 2013; Tonda et al., 2014; Ye et al., 2014; Ong et al., 2016). For example, Wang et al have reported a g-C_3_N_4_ framework, including Zn^2+^ and Fe^2+^ for the first time, which could improve the visible-light absorption, decrease the band gap (E_g_), expedite the charge mobility and extend the lifetime of charge carriers. All these characteristics are necessary to improve photocatalytic activity (Wang X. et al., 2009; Wang X. C. et al., 2009).

**Figure 4:**
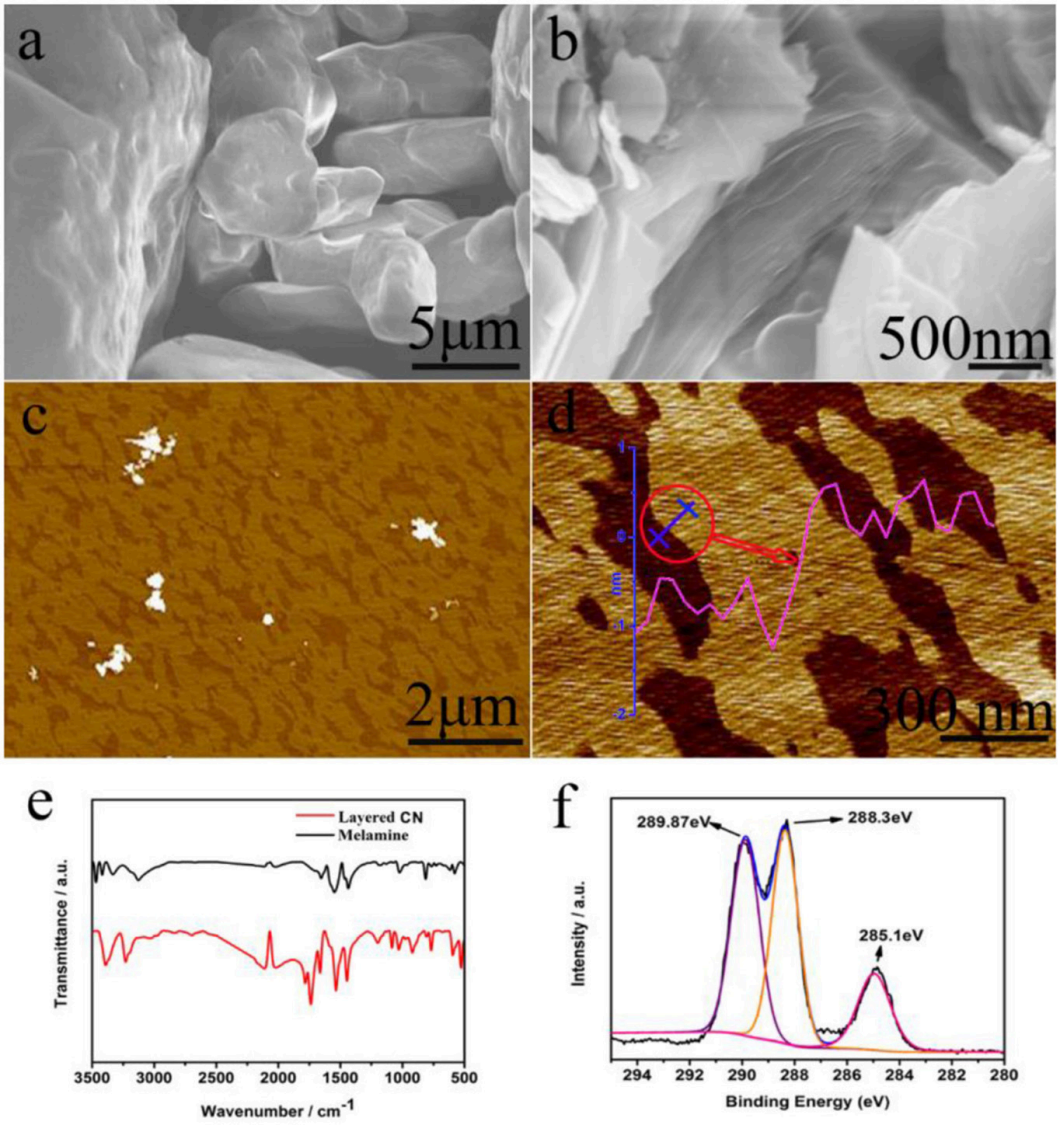
**(a)** SEM images of melamine, and **(b)** layered CN. **(c,d)** AFM images of layered CN, **(e)** Infrared spectra of melamine and synthetic layered CN, **(f)** XPS spectra of layered CN (C 1s) (Zhao G. et al., 2018). Copyright 2018, Wiley-VCH.

Since Wang et al. proposed the preparation of g-C_3_N_4_ as an efficient photocatalyst (Wang X. C. et al., 2009), g-C_3_N_4_ materials have gradually become a hot topic in the field of energy and catalysis research, due to advantages such as its low-cost, sustainability and visible-light response (Martin et al., 2014a; Xu et al., 2015; Zheng et al., 2015; Kang et al., 2016; Li J. et al., 2016). In recent years, high-efficiency 2D g-C_3_N_4_ nanosheet photocatalysts have been prepared by an organic reaction. For example, phenylene groups can be part of carbon nitrides through the copolymerization of 2-aminobenzonitrile (CN-ABN_0.5_) with dicyandiamide (Zhang et al., 2010; Zhang J. S. et al., 2012). The optical absorption edge of carbon nitride red-shifted to 700 nm from that of the pristine carbon nitride (460 nm), as the 2-aminobenzonitrile content increased. The sample (CN-ABN_0.05_ with a platinum co-catalyst) showed the topmost photocatalytic evolution of hydrogen (147 μmol h^−1^) compared with pristine carbon nitride (18 μmol h^−1^ at λ > 420 nm) (Zhang et al., 2010; Zhang J. S. et al., 2012). Zhao et al. designed a 2D g-C_3_N_4_ organic material (with a thickness of about 1.5 nm), which was successfully synthesized from melamine raw materials for the first time. The synthetic method for the 2D g-C_3_N_4_ organic material was simple and efficient. Based on the organic synthesis theory, the synthetic mechanism was theoretically explored (Figure 4, Zhao G. et al., 2018). These photocatalysts have good photocatalytic hydrogen production compared to common bulk g-C_3_N_4_ (Zhao G. et al., 2018).

## The g-C_3_N_4_ and g-C_3_N_4_-Based Nanocomposites

Two-dimensional g-C_3_N_4_ with atomic thickness has become a fascinating material in photocatalysis, because of the large specific surface area and efficiently photoexcited carriers, which can decrease the possibility of electron-hole recombination (Zhu et al., 2010; Shiraishi et al., 2014, 2015; Liu et al., 2015a,b; Shi et al., 2015). However, the synthesis or exfoliation of ultrathin (monolayer or bilayer) 2D g-C_3_N_4_ nanosheets with a homogeneous thickness, continues to be a large-scale challenge.

**Figure 5:**
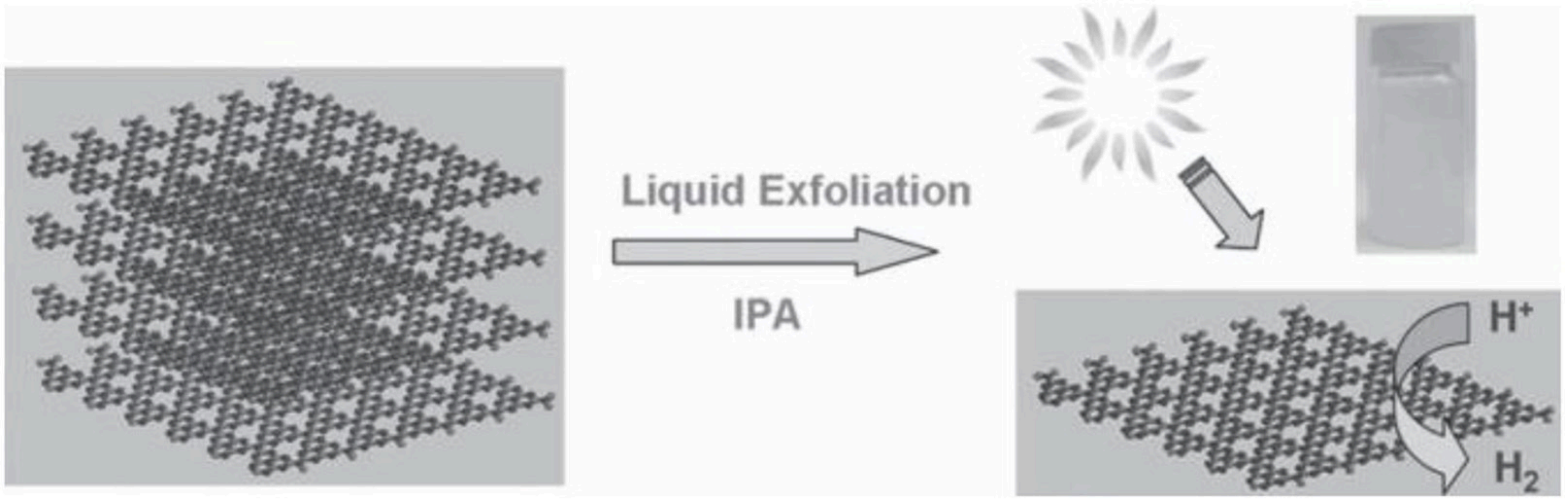
Fabrication of 2D g-C_3_N_4_ nanosheets using a simple method from bulk g-C_3_N_4_ powders for hydrogen evolution under visible light (Yang et al., 2013). Copyright 2013, Wiley-VCH.

It is known that g-C_3_N_4_ has a two-dimensional laminated structure parallel to graphene and the theoretical specific surface area of the ideal monolayer g-C_3_N_4_ can reach up to 2,500 m^2^ g^−1^. Inspired by the formation of graphene from graphite exfoliation, many effective ways have been explored for the exfoliation of raw bulk g-C_3_N_4_ to obtain a 2D ultrathin structure, such as ultrasonic liquid exfoliation, chemical exfoliation, and thermal oxidation exfoliation as well as other methods (Niu et al., 2012; Yang et al., 2013; Feng et al., 2016). For example, Yang et al. prepared g-C_3_N_4_ nanosheets from bulk g-C_3_N_4_ powders with a simple and cost-effective liquid exfoliation method (Figure 5, Yang et al., 2013). These nanosheets possess the structural features of homogeneous decentralized carbon and nitrogen atoms, an infinitesimal thickness, a large specific surface area (BET) and an optimal bandgap, which can bring about good photocatalytic activity with regards to the hydrogen evolution in visible light (Yang et al., 2013).

**Figure 6:**
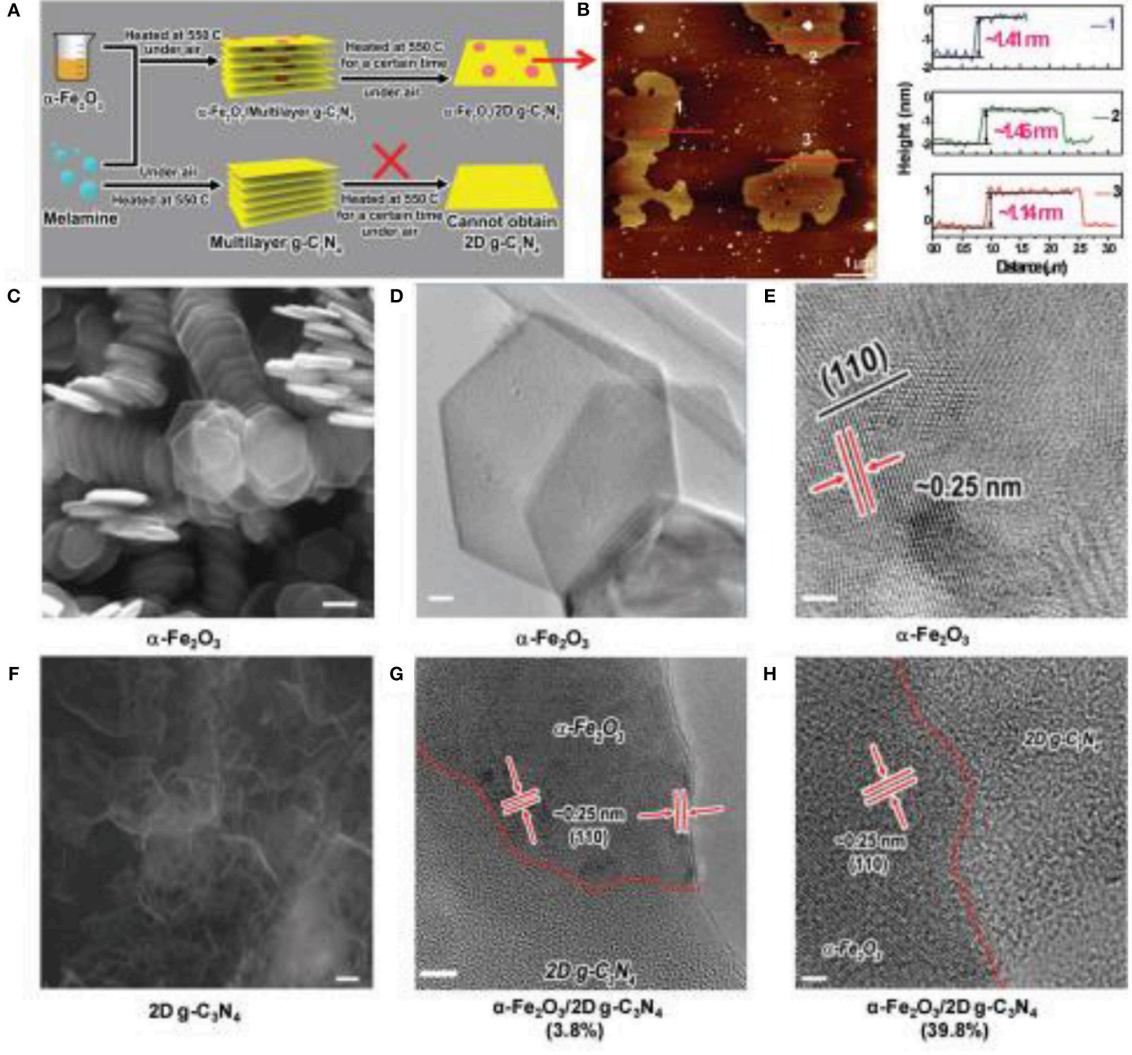
Synthesis of hybrid nanostructures. **(A)** Schematic diagram of synthesis of α-Fe_2_O_3_/2D g-C_3_N_4_ hybrid structure. **(B)** AFM image of 2D g-C_3_N_4_. **(C)** SEM image of α-Fe_2_O_3_ nanosheets (hexagonal structure). Scale bar: 100 nm. **(D)** TEM image of α-Fe_2_O_3_ nanosheet. **(E)** HRTEM image of α-Fe_2_O_3_ nanosheet. **(F)** SEM image of the 2D g-C_3_N_4_. Scale bar: 100 nm. **(G)** HRTEM image of α-Fe_2_O_3_/2D g-C_3_N_4_ (3.8%) hybrid structure, Scale bar: 5 nm. **(H)** HRTEM image of α-Fe_2_O_3_/2D g-C_3_N_4_ (39.8%) hybrid. Scale bar: 2 nm (She et al., 2017). Copyright 2017, Wiley-VCH.

Although these methods can effectively synthesize some g-C_3_N_4_ nanosheets and improve the photocatalytic property, the recombination of the electron-hole on the surfaces of the 2D materials, remains a key issue for most single-phase photocatalysts (Dong et al., 2013; Martin et al., 2014b; Ye et al., 2015). Therefore, the concept of 2D g-C_3_N_4_-based nanocomposites was proposed. Theoretical models have predicted that the restoration of photo-generated electrons/holes could be pounding down because of their effective spatial isolation on the heterojunction interface (Dong et al., 2013). Additionally, other advantages of photocatalytic reactions can also be achieved such a: good visible-light absorption and outstanding surface reaction activity. Herein, the design of 2D g-C_3_N_4_-based nanocomposites has become a research hotspot to improve the photocatalytic performance (Iwase et al., 2011; Lin and Wang, 2014; Chen et al., 2015; Han et al., 2016; She et al., 2016, 2017). For example, She et al reported that small amounts of α-Fe_2_O_3_ nanosheets could actively promote the exfoliation of g-C_3_N_4_, preparing a 2D hybrid structure that exhibited an effective Z-scheme junction (She et al., 2017). The nanostructured hybrids presented a high H_2_ evolution rate >3 × 10^4^ μmol g^−1^ h^−1^ and the quantum efficiency was about 44.35% at 420 nm, which is the uppermost value reported so far for g-C_3_N_4_ photocatalysts (Figure 6, She et al., 2017).

**Figure 7:**
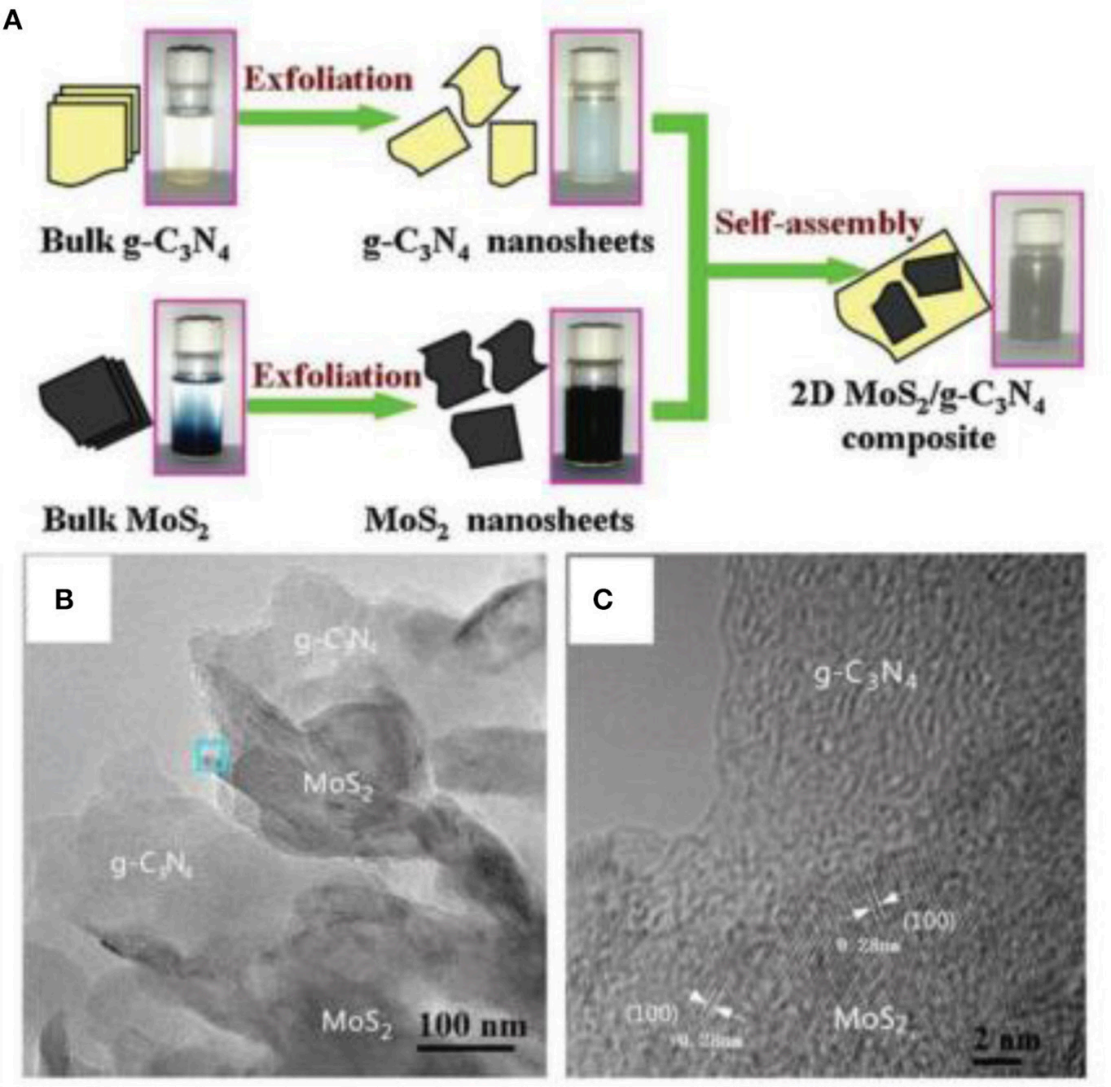
**(A)** Schematic diagram of 2D MoS_2_/g-C_3_N_4_ nanocomposite preparation. **(B)** TEM and **(C)** HRTEM images of 2D MoS_2_/g-C_3_N_4_ nanocomposite (Li X. G. et al., 2016). Copyright 2016, Elsevier.

A layered-structure, MoS_2_, is also a candidate for incorporation with g-C_3_N_4_ to construct 2D/2D nanocomposites (Hou et al., 2013; Li X. G. et al., 2016). For example, Li et al. designed a 2D g-C_3_N_4_ and MoS_2_ heterojunction via means of the self-assembly of 2D g-C_3_N_4_ with MoS_2_ nanosheets (Li X. G. et al., 2016). As shown in Figure 7, the 2D g-C_3_N_4_ and MoS_2_ nanosheets were prepared from the exfoliation of bulk g-C_3_N_4_ and MoS_2_ raw materials, through ultrasonication (Li X. G. et al., 2016). Thin g-C_3_N_4_ and MoS_2_ 2D nanosheets were observed in 2D g-C_3_N_4_/MoS_2_ nanocomposites by TEM images (Figure 7). This type of g-C_3_N_4_/ MoS_2_ photocatalysts also showed a good photocatalytic effect.

## Mechanism of a 2D g-C_3_N_4_ Photocatalyst System

**Figure 8:**
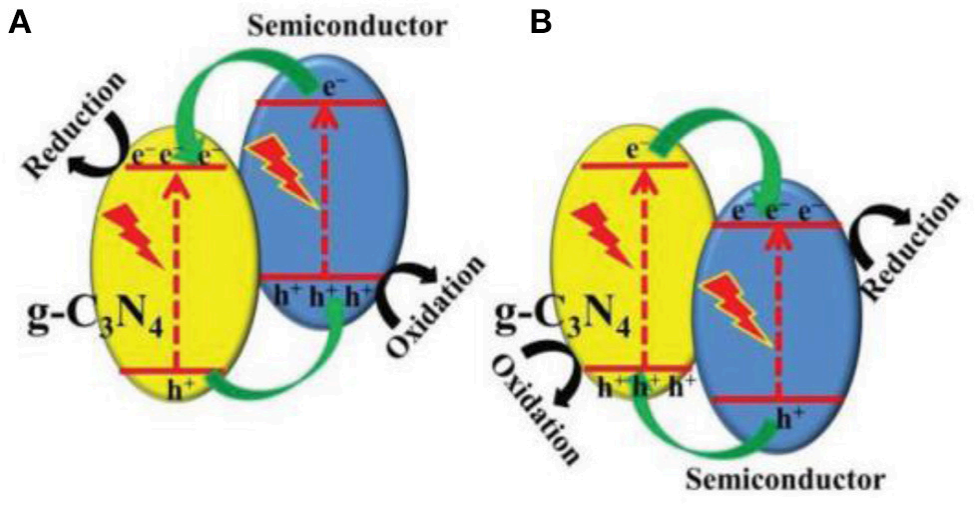
**(A,B)** Charge transfer in the conventional type-II g-C_3_N_4_-based heterojunction systems (Fu et al., 2017). Copyright 2017, Wiley-VCH.

In a single 2D g-C_3_N_4_ system, the photo-excited electrons of the conduction band (CB) generally return to the valence band (VB) (Tian et al., 2014), while the unpopular recovery of photo-generated electrons and holes are a great disadvantage of photocatalytic reactions (Yin et al., 2016). The photocatalyst is used as a semiconductor, to intimately constitute with g-C_3_N_4_, to create a suitable band structure. The spatial isolation of photo-generated electrons and holes can be realized through an effective charge transfer on the two semiconductor interfaces (Figure 8, Jiang et al., 2013; Liu et al., 2016; Zhang X. J. et al., 2016; Fu et al., 2017). Commonly, the bandgap of pristine g-C_3_N_4_ bandgap is about 2.7 eV and their CB and VB are situated at −1.1 eV and +1.6 eV, respectively (Cao et al., 2015). g-C_3_N_4_ is therefore used as a photocatalyst for photo-reduction reactions, because of its sufficiently negative conduction band position in Figure 8. Generally, 2D g-C_3_N_4_-based hetero-junction systems are very effective in separating photo-generated electron/hole pairs, because of the component photocatalyst has this kind of Z-Z band structures (Cao et al., 2015). Therefore, an appropriate band-structure is important to consider when choosing the component photocatalyst for the structuring of 2D g-C_3_N_4_-based heterojunction photocatalysts (Fu et al., 2017).

**Figure 9:**
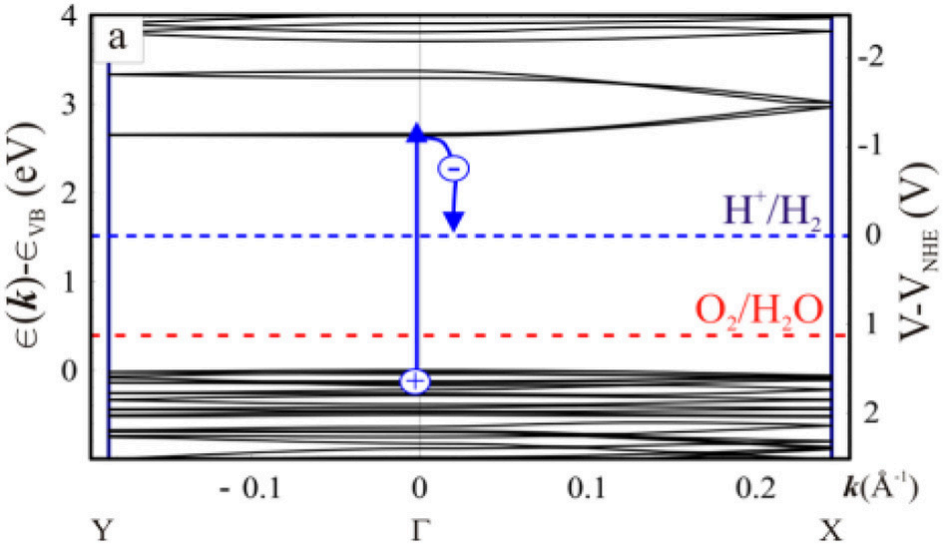
Density functional theory of band structure (Thomas et al., 2008). Copyright 2008, Royal Society of Chemistry.

**Figure 10:**
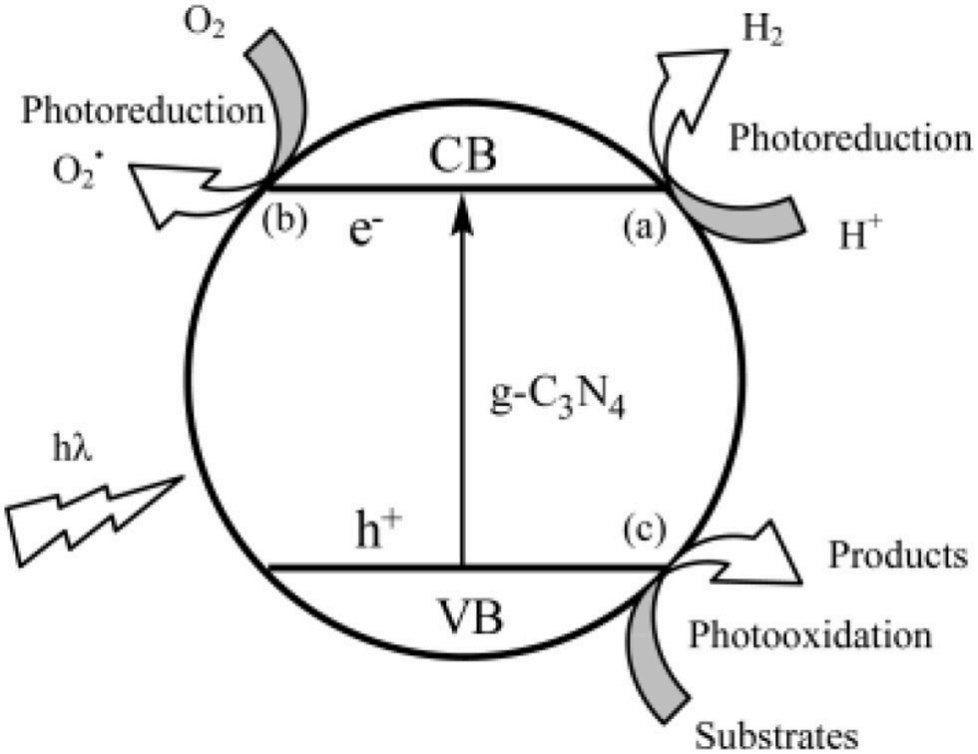
Photocatalytic mechanism of g-C_3_N_4_ photocatalystt (Wang et al., 2012). Copyright 2012, American Chemical Society.

Additionally, the band gap requires that the oxidation of the photo-generated hole has enough strength, in order to obtain oxygen from the oxidation of water, and the photo-generated electron must restore enough, to reduce the water, in order to yield H_2_ (Li et al., 2012). In other words, the location of the HOMO-LUMO band must consume the water oxidation-reduction potential (Wang et al., 2012). As illustrated in Figure 9, it is able to run half of two independent reactions, by calculating the carbon nitride band positions (Thomas et al., 2008; Maeda et al., 2014). The type of containment in an organic semiconductor is a rare condition in Figure 10 (Wang et al., 2012).

**Figure 11:**
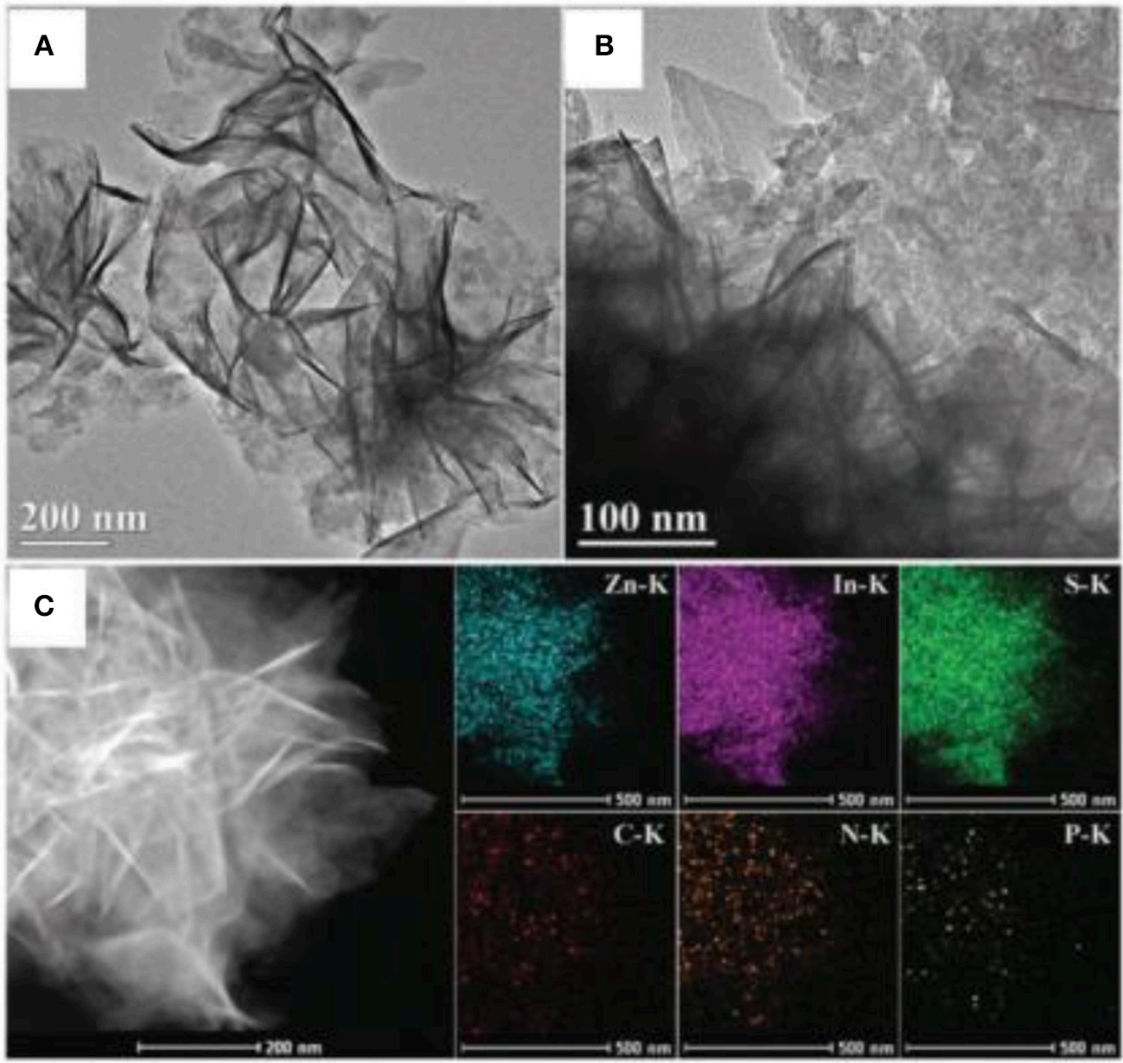
**(A,B)** TEM images, and **(C)** EDS mapping images of P-C_3_N_4_/ZnIn_2_S_4_ nanocomposites (Chen et al., 2016). Copyright 2016, Royal Society of Chemistry.

**Figure 12:**
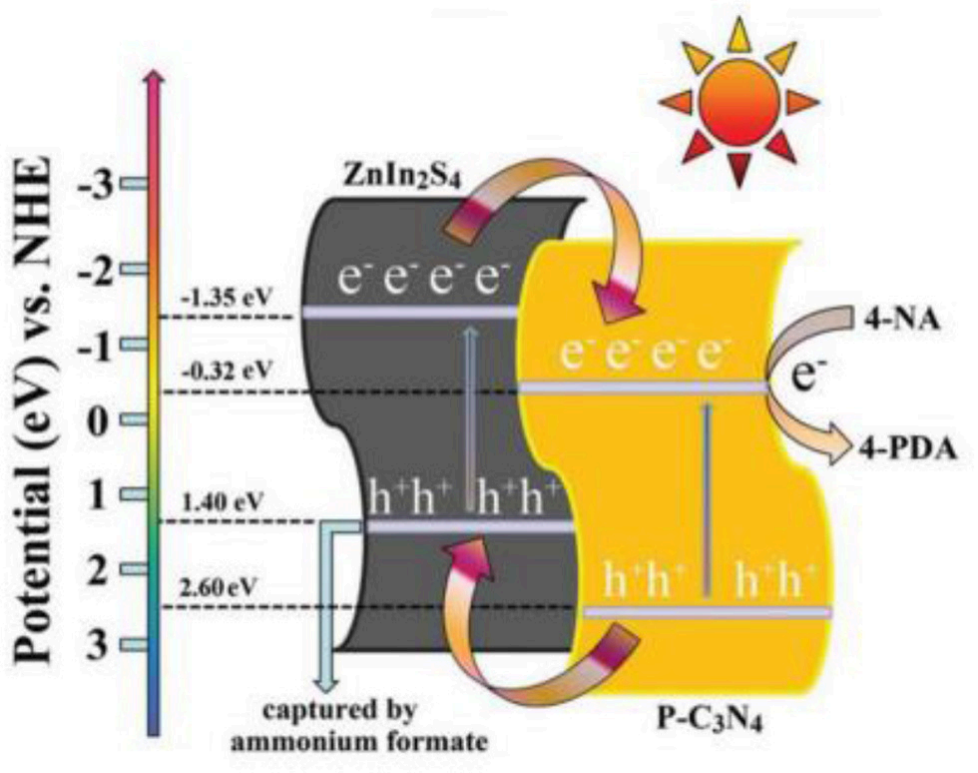
Water photolysis mechanism of P-C3N4/ZnIn2S4 photocatalyst under simulated solar irradiation (Chen et al., 2016). Copyright 2016, Royal Society of Chemistry.

In 2016, Chen et al. fabricated a 2D/2D P-doped g-C_3_N_4_/ZnIn_2_S_4_ photocatalyst by an *in situ* loading method, wherein ZnIn_2_S_4_ nanosheets where grown on the P-doped mesoporous g-C_3_N_4_ nanosheet surface (Chen et al., 2016). As shown in Figure 11. the 2D nanosheet structure can clearly be observed for the P-C_3_N_4_/ZnIn_2_S_4_ nanocomposites. Moreover, the EDS mapping images of the P-C_3_N_4_/ZnIn_2_S_4_ show that all the elements (Zn, In, S, C, N, and P) are evenly dispersed on the surface of the photocatalyst (Chen et al., 2016). This type of a special 2D/2D surface contact can provide more contact areas between P-C_3_N_4_ and ZnIn_2_S_4_, which is conducive to an effective charge carrier separation. Under light irradiation, the photo-generated electrons can transfer from the CB of ZnIn_2_S_4_ to the CB of P-C_3_N_4_. Similarly, the photo-generated holes can shift from the VB of P-C_3_N_4_ to the VB of ZnIn_2_S_4_, as shown in Figure 12. The spatial isolation of photo-generated charge carriers can vastly optimize the catalytic performance of the P-C_3_N_4_/ZnIn_2_S_4_ photocatalyst (Chen et al., 2016).

## Summary and Outlook

Currently, two-dimensional g-C_3_N_4_, a metal-free and visible-light-responsive photocatalyst, in the field of hydrogen-production through water splitting, is a hot topic in research. This critical review summarizes the ultramodern progress in the design and preparation of 2D g-C_3_N_4_ and g-C_3_N_4_-based composites. Although significant advances in 2D g-C_3_N_4_-based photocatalysts have been made, photocatalytic efficiency remains too low. However, its wide application proves that 2D g-C_3_N_4_-based photocatalysts are prospective materials in the practical application of efficient sun-energy conversion in the future.

## Author Contributions

All authors listed have made a substantial, direct and intellectual contribution to the work, and approved it for publication.

### Conflict of interest statement

The authors declare that the research was conducted in the absence of any commercial or financial relationships that could be construed as a potential conflict of interest.
